# Workplace infection prevention control measures and work engagement during the COVID‐19 pandemic among Japanese workers: A prospective cohort study

**DOI:** 10.1002/1348-9585.12350

**Published:** 2022-08-08

**Authors:** Kazunori Ikegami, Hajime Ando, Yoshihisa Fujino, Hisashi Eguchi, Keiji Muramatsu, Tomohisa Nagata, Seiichiro Tateishi, Mayumi Tsuji, Akira Ogami, for the CORoNaWork project

**Affiliations:** ^1^ Department of Work Systems and Health Institute of Industrial Ecological Sciences, University of Occupational and Environmental Health Kitakyushu Japan; ^2^ Department of Environmental Epidemiology Institute of Industrial Ecological Sciences, University of Occupational and Environmental Health Kitakyushu Japan; ^3^ Department of Mental Health Institute of Industrial Ecological Sciences, University of Occupational and Environmental Health Kitakyushu Japan; ^4^ Department of Preventive Medicine and Community Health, School of Medicine University of Occupational and Environmental Health Kitakyushu Japan; ^5^ Department of Occupational Health Practice and Management Institute of Industrial Ecological Sciences, University of Occupational and Environmental Health Kitakyushu Japan; ^6^ Disaster Occupational Health Center Institute of Industrial Ecological Sciences, University of Occupational and Environmental Health Kitakyushu Japan; ^7^ Department of Environmental Health, School of Medicine University of Occupational and Environmental Health Kitakyushu Japan

**Keywords:** COVID‐19, infection control measures, infection prevention, work engagement, workplace

## Abstract

**Objectives:**

Our objective was to assess the effect of appropriate workplace IPC measures on employees' work engagement. It could be important to note how workplace infection prevention control (IPC) measures for COVID‐19 contribute to positive mental health among workers. We hypothesized that if workplace IPC measures are adequately implemented, they would have a positive effect on employees' work engagement.

**Methods:**

We conducted an internet‐based prospective cohort study from December 2020 (baseline) to December 2021 (follow‐up after 1 year) using self‐administered questionnaires. At baseline, 27036 workers completed the questionnaires, while 18 560 (68.7%) participated in the one‐year follow‐up. After excluding the 6578 participants who changed jobs or retired during the survey period, or telecommuted more than 4 days per week, 11 982 participants were analyzed. We asked participants about the implementation of workplace IPC measures at baseline and conducted a follow‐up using a nine‐item version of the Utrecht Work Engagement Scale (UWES‐9).

**Results:**

Four groups were created according to the number of workplace IPC measures implemented. The mean (SD) UWES‐9 score of the “0–2” group was the lowest at 18.3 (13.2), while that of the “8” group was the highest at 22.6 (12.6). The scores of the “3–5,” “6–7,” and “8” groups were significantly higher than that of the “0–2” group (all, *p* < .001). The p trend of the four groups was also significant (*p* < .001).

**Conclusions:**

Promoting workplace IPC measures improves workers' work engagement, and a dose–response relationship exists between workplace IPC measures and work engagement.

## 1 INTRODUCTION

The coronavirus disease (COVID‐19), which was caused by severe acute respiratory syndrome coronavirus 2 (SARS‐CoV‐2) and broke out in December 2019, has caused a pandemic worldwide due to its viral mutations and is yet to be fully contained.^1^, ^2^ In Japan, a COVID‐19 pandemic occurred after 2020, and the Japanese government repeatedly declared a state or quasi‐state of emergency; focused on anti‐infection measures; and strengthened infection control measures such as non‐pharmaceutical interventions (NPIs). In addition, COVID‐19 vaccination was also promoted both in the community and in occupational fields. However, in November 2021, the SARS‐CoV‐2 omicron variant (B.1.1.529) was classified as a variant of concern (VOC); by March 2022, the omicron variant had continued to spread COVID‐19 worldwide, including in Japan. Hence, the infection prevention control (IPC) of COVID‐19 has been an important issue.^3^

COVID‐19 is thought to be transmitted mainly by droplets containing the virus (droplet infection).^4^ However, it has been reported that droplet nuclei (aerosols), which are transformed when droplets float in the air, can also cause COVID‐19 infection.^5^, ^6^ Various NPI strategies have been implemented to reduce SARS‐CoV‐2 transmission, including mask‐wearing, hand hygiene, physical distancing, and proper room ventilation.^7^ In particular, the workplace is considered one of the most likely places for the spread of COVID‐19 because many employees work and communicate in the same space.

In Japan, workplace IPC measures are one of the most important issues in fulfilling a company's obligation to promote employees' health and safety and ensure business continuity amidst the COVID‐19 epidemic. To this end, many guidelines and checklists for workplace IPC measures have been published, including “A Guide for Businesses and Employers Responding to Novel Coronavirus Disease (COVID‐19),” which was published by the Japanese Society for Occupational Health (JSOH).^8^ Based on these guidelines, workplace IPC measures have been implemented in many companies. Indeed, in addition to basic measures such as physical distancing, wearing masks, and washing hands, other proposed measures include enhancing office room ventilation; refraining from or restricting business trips, visitors, social gatherings, and face‐to‐face meetings; setting up partitions; daily physical condition checks; and promoting sick leave when employees feel ill.^8^

It has been widely reported that the COVID‐19 pandemic may have an important psychological influence on people.^9^ We are particularly interested in the impact of workplace IPC measures on workers' mental health. In general, work environment, work organization, and work‐related behaviors are considered to be factors that influence workers' mental health, psychological distress, and well‐being.^10^ There are also reports on the COVID‐19 pandemic and work stress. For example, it has been reported that anxiety about COVID‐19 infection in the workplace may enhance job demands and psychological distress.^11^ It has likewise been reported that telecommuting, which is implemented as a COVID‐19 IPC measure, has a positive impact on workers' work engagement.^12^ Thus, it is important to clarify how workplace environmental factors and work‐related behaviors affect workers' mental health during the COVID‐19 pandemic.

In recent years, mental health support for workers has come to be regarded as important—not only in preventing workers' mental disorders and resolving their mental problems but also in promoting the revitalization of both workers and company organizations.^13^ Recent research in the field of occupational health has focused on themes involving positive mental health, such as improving well‐being and productivity, as well as themes involving negative mental health, such as reducing job stress and treating depression.^14^ One of the leading indicators of positive mental health among workers that have been attracting attention is work engagement.

Work engagement has been defined as “a positive, fulfilling, work‐related state of mind that is characterized by vigor, dedication, and absorption.”^15^, ^16^ Employees with high work engagement are considered to be physically and mentally healthy, energetic, enthusiastic, and productive.^15^, ^17^ Work engagement can be easily assessed using questionnaires, and one such well‐known questionnaire is the Utrecht Work Engagement Scale (UWES) developed by Schaufeli et al. The UWES has been standardized in many countries and has been confirmed to have good results in terms of reliability and validity.^15^ A Japanese version of UWES, which was developed by Shimazu et al.,^18^ has been used in many studies.

We hypothesized that if workplace IPC measures are adequately implemented, they would have a positive effect on employees' work engagement. Therefore, in this study, we prospectively evaluated the influence of workplace IPC measures on workers' work engagement by analyzing data from the Collaborative Online Research on the Novel‐coronavirus and Work (CORoNaWork) Project. Furthermore, we analyzed which workplace IPC measures have a strong influence on workers' work engagement.

## 2 METHODS

### 2.1 Study design and setting

This study is a prospective cohort study conducted from December 2020 (baseline survey) to December 2021 (follow‐up survey). Both the baseline and follow‐up surveys were conducted using self‐administered questionnaires on the Internet. All participants gave informed consent to participate in the study. The study was approved by the ethics committee of the University of Occupational and Environmental Health, Japan (reference nos. R2‐079 and R3‐006). The study protocol of the CORoNaWork study, including the sampling plan and subject recruitment procedure according to the Checklist for Reporting Results of Internet E‐Surveys (CHERRIES) Checklist, has been reported in our previous work.^19^, ^20^, ^21^

The baseline survey was conducted when Japan was on maximum alert levels at the beginning of the third wave of COVID‐19, as the number of COVID‐19 infections and deaths were overwhelmingly higher in the third wave than in the first and second. The follow‐up survey was conducted when the fifth wave had settled down and the number of infections was decreasing.

### 2.2 Participants

#### 2.2.1 Baseline survey

The target population comprised subjects between the ages of 20 and 65 who were working at the time of the baseline survey. Sampling was conducted with allocation by region, occupation, and sex. Regions were divided into five levels of 47 prefectures according to the level of infection. Occupations were likewise divided into office workers and non‐office workers. Thus, a total of 20 blocks of 5 regions, 2 occupations, and 2 sexes were assigned, and each block was sampled in equal numbers. We planned to study 30 000 people overall, and thus attempted to gain at least 1500 participants in each block.

The survey was commissioned by Cross Marketing Inc. (Tokyo, Japan). Of their 4.7 million pre‐registered monitors, approximately 600 000 were sent an email request to participate in the survey. Of these, 55 045 participated in the initial screening survey, while 33 087 met the inclusion criteria for the same.

**FIGURE 1:**
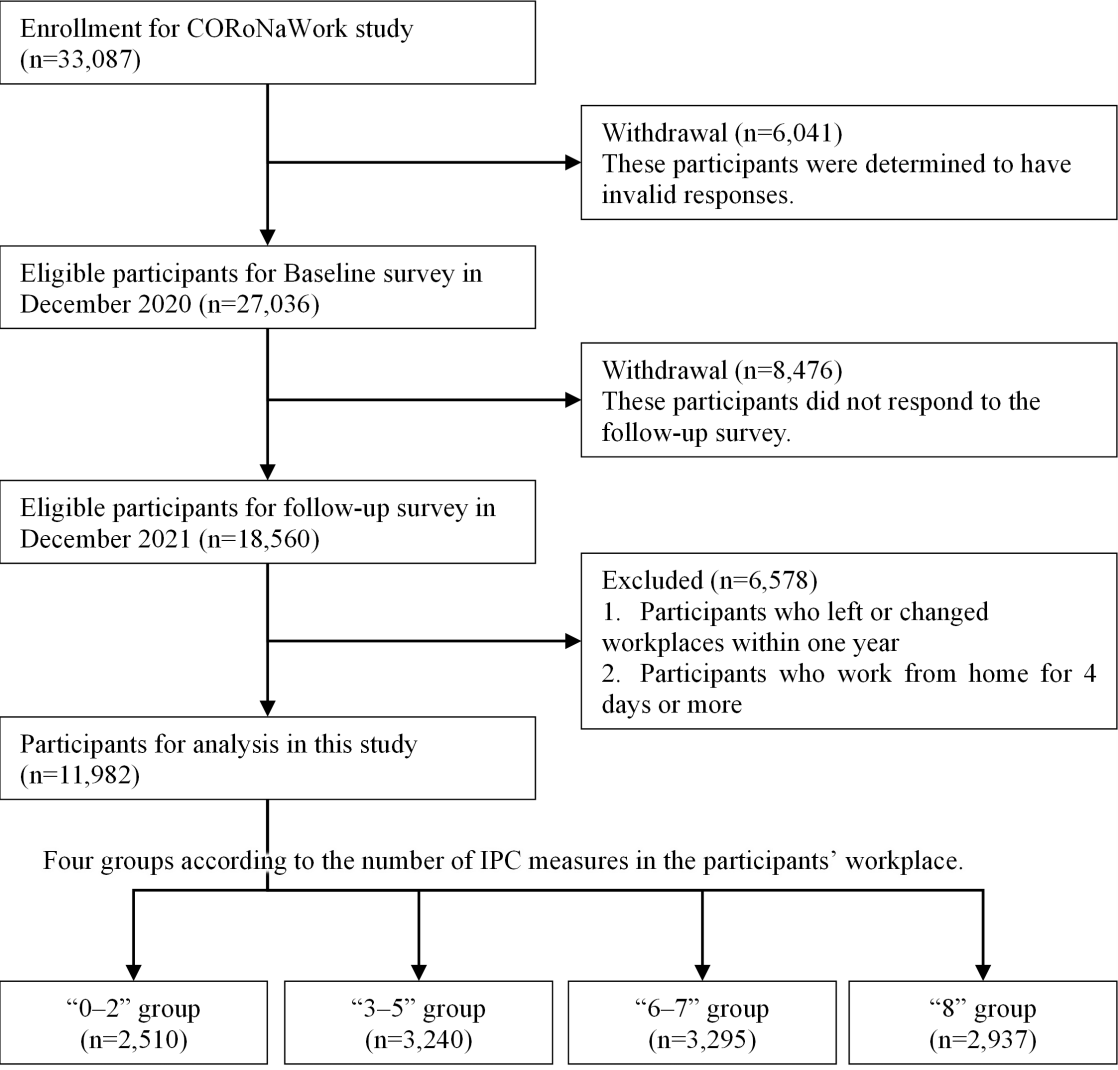
Flowchart of the study population selection.

Of the 33 087 initial participants, 27 036 (excluding those judged as fraudulent responses) were included in this analysis. The following criteria (i.e., the exclusion criteria) were used to determine fraudulent responses: extremely short response time (≤6 minutes), extremely low body weight (<30 kg), extremely short height (<140 cm), inconsistent answers to similar questions throughout the survey (e.g., inconsistency to questions about marital status and living situation), and wrong answers to a staged question used to identify fraudulent responses (choose the third‐largest number from five numbers) (Figure 1).

#### 2.2.2 Follow‐up survey

A follow‐up survey was conducted in December 2021, 1 year after the baseline survey. A total of 18 560 (tracking rate: 68.7%) respondents participated in the follow‐up survey. We excluded 6578 participants who changed jobs or retired during the survey period and those who telecommuted for more than 4 days per week (i.e., those who rarely worked in the workplace). Ultimately, 11 982 participants were analyzed (Figure 1).

### 2.3 Evaluation of work engagement

A nine‐item version of the UWES was used to measure work engagement.^22^ The Japanese version of the UWES‐9 has been verified for reliability and validity by Shimazu et al.^23^ Each question item consists of a seven‐point Likert scale ranging from 0 for “never” to 6 for “always.” The UWES‐9 calculates three subscales (vitality, enthusiasm, and immersion), consisting of three items each, in addition to the total score. Higher scores indicate a higher state of work engagement. The score range of the UWES‐9 is 0–54, and the range of each subscale is 0–18. In the present sample, the Cronbach's alpha of UWES‐9 (total score), vigor, dedication, and absorption were 0.97, 0.93, 0.91, and 0.92, respectively.

### 2.4 Evaluation of workplace IPC measures for COVID‐19

An original list of workplace IPC measures for COVID‐19 was developed. First, we prepared an initial list of workplace IPC measures based on the relevant publications listing standard workplace IPC measures in Japan.^8^, ^24^, ^25^ Subsequently, a draft list was prepared in consultation with an expert panel based on their practical experience. Finally, we identified nine priority items of workplace IPC Measures with the approval of all study collaborators and developed a final list. The list of original workplace IPC measures consisted of eight measures in the workplace and one measure related to telecommuting.^26^

We asked the participants to answer with a “yes” or “no” as to whether or not the following eight workplace IPC measures, with the exception of the item related to telecommuting, for COVID‐19 had been implemented by their workplace at baseline: (1) placing restrictions on business trips or going out for business (stopping business trips); (2) refraining from and placing restrictions on visitors (arranging health screenings for visitors); (3) refraining from or requesting a limit on the number of people at social gatherings and dinners (restricting work‐related social gatherings and entertainment); (4) refraining from or limiting face‐to‐face internal meetings (restricting face‐to‐face meetings); (5) wearing masks at all times during work hours (encouraging mask‐wearing at work); (6) installing partitions and revising the workplace layout (installing partitions or changing the working environment); (7) recommending workers perform daily temperature checks at home (enforcing temperature measurement); and (8) requesting employees not to come to work when they are not feeling well (requesting that employees refrain from going to work when ill). In addition, variables regarding the eight items were calculated by totaling the number of “yes” responses for each participant (range: 0–8) to evaluate based on the number of workplace IPC measures.

### 2.5 Outcome and measurements

The participants' UWES‐9 scores in the follow‐up survey were used as outcome variables. The participants were divided into four groups (“0–2,” “3–5,” “6–7,” and “8”) according to the number of workplace IPC measures implemented in their workplace at baseline, and these were used as exposure variables. To avoid bias in the participants' distribution of these four groups, the groups were organized with reference to the quartiles of the number of workplace IPC measures. The participants' distribution (%) was 21.0% in the “0–2” group, 27.0% in the “3–5” group, 27.5% in the “6–7” group, and 24.5% in the “8” group. In an additional analysis, each item of the eight workplace IPC measures was used as an exposure variable.

Sex, age (20–29 years, 30–39 years, 40–49 years, 50–59 years, ≥60 years), educational background (middle school/high school, junior college/vocational school, university/graduate school), number of household members (1 person, 2 people, 3 people, ≥4 people), standard industrial classification (primary industry, secondary industry, tertiary industry), job type (regular employee, managers, others), and size of the workplace (1–9 employees, 10–49 employees, 50–999 employees, ≥1000 employees) were used as confounders. The standard industrial classification was defined by the Japanese Ministry of Internal Affairs and Communications.

These variables, except for the size of the workplace, were collected in the baseline survey. While data on the size of the participants' companies were collected in the baseline survey, the size of the workplace was likewise asked in the follow‐up survey to obtain more detailed information.

### 2.6 Statistical analyses

To estimate whether the workplace IPC measures were associated with work engagement among the participants, we used a multilevel regression analysis nested in the prefecture of residence in order to account for regional variability. An age‐sex adjusted model and multivariate‐adjusted model were estimated. Both models included age, sex, education, number of household members, and the four groups according to the number of COVID‐19 infection control measures in the workplace as the fixed effects, while the prefecture of residence was the random effect. In addition, the p‐values of multilevel regression analysis were calculated by considering each category scale of the number of workplace IPC measures as continuous variables (*p* for trend).

In all tests, the threshold for significance was set at *p* < .05. Stata/SE Ver.15.1 (StataCorp LLC, College Station, Texas, United States) was used for the analysis.

## 3 RESULTS

### 3.1 Participants and descriptive data

Compared to the participants working in places with fewer workplace IPC measures, the participants working in companies with more workplace IPC measures tended to have higher education and be married. Smaller‐sized workplaces tended to have fewer workplace IPC measures, while larger‐sized workplaces tended to have more. In terms of standard industry classification, the primary industry had the highest proportion of workplaces implementing “0–2” workplace IPC measures among the three industries, and the secondary industry had the highest proportion of workplaces implementing 8 workplace IPC measures. The tertiary industry had the highest proportion of workplaces implementing “3–5” and “6–7” workplace IPC measures (Table 1).

**TABLE 1:** Characteristics of each group according to the number of workplace IPC measures for COVID‐19 at baseline

Item	Group by # of workplace IPC measures							
	“0–2” group		“3–5” group		“6–7” group		“8” group	
	*n* = 2510		*n* = 3240		*n* = 3295		*n* = 2937	
	*n*	(%)	*n*	(%)	*n*	(%)	*n*	(%)
Sex, male	1507	(60.0)	1764	(54.4)	1843	(55.9)	1686	(57.4)
Age								
20–29 years	93	(3.7)	168	(5.2)	159	(4.8)	159	(5.4)
30–39 years	374	(14.9)	511	(15.8)	522	(15.8)	473	(16.1)
40–49 years	839	(33.4)	1068	(33.0)	1039	(31.5)	886	(30.2)
50–59 years	899	(35.8)	1163	(35.9)	1261	(38.3)	1122	(38.2)
≥60 years	305	(12.2)	330	(10.2)	314	(9.5)	297	(10.1)
Education								
Junior high/high school	970	(38.6)	923	(28.5)	825	(25.0)	614	(20.9)
Vocational school/college	609	(24.3)	763	(23.5)	686	(20.8)	624	(21.2)
University/graduate school	931	(37.1)	1554	(48.0)	1784	(54.1)	1699	(57.8)
Size of workplace^a^								
≤9 employees	1392	(55.5)	995	(30.7)	578	(17.5)	330	(11.2)
10–49 employees	664	(26.5)	1121	(34.6)	1027	(31.2)	682	(23.2)
50–999 employees	381	(15.2)	943	(29.1)	1357	(41.2)	1416	(48.2)
≥1000 employees	73	(2.9)	181	(5.6)	333	(10.1)	509	(17.3)
Standard industrial classification^b^								
Primary industry	65	(2.6)	26	(0.8)	13	(0.4)	7	(0.2)
Secondary industry	759	(30.2)	693	(21.4)	779	(23.6)	919	(31.3)
Tertiary industry	1686	(67.2)	2521	(77.8)	2503	(76.0)	2011	(68.5)
Job type								
Regular employee	1289	(51.4)	1488	(45.9)	1485	(45.1)	1383	(47.1)
Manager	138	(5.5)	280	(8.6)	430	(13.1)	500	(17.0)
Other	1083	(43.1)	1472	(45.4)	1380	(41.9)	1054	(35.9)
Marital status, unmarried	1203	(47.9)	1462	(45.1)	1336	(40.5)	1087	(37.0)
Number of household members								
1 person	523	(20.8)	644	(19.9)	638	(19.4)	520	(17.7)
2 people	710	(28.3)	883	(27.3)	883	(26.8)	728	(24.8)
3 people	637	(25.4)	844	(26.0)	827	(25.1)	758	(25.8)
≥4 people	640	(25.5)	869	(26.8)	947	(28.7)	931	(31.7)

Abbreviation: IPC, infection prevention control.

^a^ This standard industrial classification was defined by the Japanese Ministry of Internal Affairs and Communications.

^b^ Although the data on the size of the companies where the participants worked was collected in the baseline survey, the size of the workplace was also collected in the follow‐up survey to obtain more detailed information.

### 3.2 UWES‐9 and workplace IPC measures

As for the mean (*SD*), UWES‐9 scores among the four groups according to the number of workplace IPC measures, the “0–2” group had the lowest at 18.3 (13.2), and the “8” group had the highest at 22.6 (12.6). In both the sex‐age adjusted model and the multivariate model, the scores of the “3–5,” “6–7,” and “8” groups were significantly higher compared with that of the “0–2” group (all, *p* < .001). The p for trend of the four groups was also significant (*p* < .001) (Tables 2 and 3).

**TABLE 2:** Participants' UWES‐9 score of each group according to the number of workplace IPC measures for COVID‐19 at the follow‐up

UWES‐9 item	Groups by number of workplace IPC measures							
	“0–2” group		“3–5” group		“6–7” group		“8” group	
	(*n* = 2510)		(*n* = 3240)		(*n* = 3295)		(*n* = 2937)	
	Mean	(*SD*)	Mean	(*SD*)	Mean	(*SD*)	Mean	(*SD*)
Total score	18.3	(13.2)	20.8	(12.8)	21.7	(12.2)	22.6	(12.6)
Vigor	5.7	(4.5)	6.4	(4.4)	6.8	(4.3)	7.1	(4.4)
Dedication	6.7	(4.7)	7.7	(4.5)	8.0	(4.3)	8.2	(4.4)
Absorption	5.9	(4.5)	6.7	(4.5)	6.9	(4.2)	7.2	(4.3)

Abbreviation: IPC, infection prevention control; *SD*, standard deviation; UWES‐9, nine‐item version of the Utrecht Work Engagement Scale.

**TABLE 3:** Association between participants' work engagement and the number of workplace IPC measures for COVID‐19

UWES‐9 items Group by number of workplace IPC measures	Sex‐age adjusted			Multivariate		
	Coef.	[95% CI]	*p*	Coef.	[95% CI]	*p*
Total score						
“0–2” group	Ref.		<.001*	Ref.		<.001*
“3–5” group	2.59	[1.94–3.24]	<.001	2.86	[2.20–3.52]	<.001
“6–7” group	3.47	[2.82–4.12]	<.001	4.01	[3.32–4.69]	<.001
“8” group	4.34	[3.67–5.01]	<.001	5.04	[4.32–5.77]	<.001
Vigor						
“0–2” group	Ref.		<.001*	Ref.		<.001*
“3–5” group	0.76	[0.53–0.98]	<.001	0.85	[0.62–1.08]	<.001
“6–7” group	1.06	[0.84–1.29]	<.001	1.25	[1.01–1.49]	<.001
“8” group	1.43	[1.20–1.66]	<.001	1.67	[1.42–1.92]	<.001
Dedication						
“0–2” group	Ref.		<.001*	Ref.		<.001*
“3–5” group	1.02	[0.79–1.25]	<.001	1.11	[0.88–1.34]	<.001
“6–7” group	1.39	[1.16–1.62]	<.001	1.57	[1.33–1.81]	<.001
“8” group	1.57	[1.34–1.81]	<.001	1.82	[1.56–2.07]	<.001
Absorption						
“0–2” group	Ref.		<.001*	Ref.		<.001*
“3–5” group	0.81	[0.58–1.03]	<.001	0.90	[0.67–1.13]	<.001
“6–7” group	1.02	[0.79–1.24]	<.001	1.19	[0.95–1.42]	<.001
“8” group	1.34	[1.11–1.57]	<.001	1.55	[1.30–1.80]	<.001

Abbreviations: CI, confidence interval; IPC, infection prevention control; UWES‐9; nine‐item version of the Utrecht Work Engagement Scale.

* *p* for trend.

As for the mean (*SD*) subscale scores of vigor, dedication, and absorption among the four groups, the “0–2” group was again the lowest at 5.7 (4.5), 6.7 (4.7), and 5.9 (4.5), respectively. The “8” group was highest, at 7.1 (4.4), 8.2 (4.4), and 7.2 (4.3), respectively. In both the sex‐age adjusted model and the multivariate model, the scores of the “3–5,” “6–7,” and “8” groups were significantly higher than those of the “0–2” group (all, *p* < .001). The p for trend of the four groups was also significant (*p* < .001) (Tables 2 and 3).

Table 4 shows the mean scores (SD) of the four UWES‐9 items, that is, total score, vigor, dedication, and absorption in each of the workplace IPC measures items. In all workplace IPC measures items, the UWES‐9 scores were higher for those who responded that they had access to the IPC measures (“yes”), than in their counterparts. In the statistical analysis, the implementation of “requesting that employees refrain from going to work when ill” had a significant positive effect on all UWES‐9 items (all, *p* < .001) (Table 5).

**TABLE 4:** Participants' UWES‐9 score of each item of workplace IPC measures for COVID‐19 at the follow‐up

Items of workplace IPC measures	Participants' response	*n*	UWES‐9 items							
			Total score		Vigor		Dedication		Absorption	
			M	(*SD*)	M	(*SD*)	M	(*SD*)	M	(*SD*)
Stopping business trips	No	5428	28.8	(13.1)	9.2	(4.5)	10.3	(4.6)	9.3	(4.5)
	Yes	6554	30.9	(12.4)	9.8	(4.3)	11.1	(4.4)	10.0	(4.3)
Arranging health screenings for visitors	No	6651	29.1	(12.9)	9.3	(4.4)	10.4	(4.6)	9.4	(4.4)
	Yes	5331	31.0	(12.5)	9.9	(4.4)	11.1	(4.4)	10.0	(4.3)
Restricting work‐related social gatherings and entertainment	No	3364	28.0	(13.3)	8.9	(4.5)	9.9	(4.7)	9.1	(4.5)
	Yes	8618	30.7	(12.5)	9.8	(4.3)	11.0	(4.4)	9.9	(4.3)
Restricting face‐to‐face meetings	No	5508	28.8	(13.1)	9.2	(4.5)	10.3	(4.6)	9.4	(4.5)
	Yes	6474	30.9	(12.4)	9.9	(4.3)	11.1	(4.3)	10.0	(4.3)
Encouraging mask‐wearing at work	No	2414	28.0	(13.1)	9.0	(4.4)	9.9	(4.6)	9.1	(4.5)
	Yes	9568	30.4	(12.6)	9.7	(4.4)	10.9	(4.4)	9.8	(4.4)
Installing partitions or changing the working environment	No	4862	28.7	(12.9)	9.1	(4.4)	10.2	(4.6)	9.3	(4.4)
	Yes	7120	30.8	(12.6)	9.8	(4.4)	11.0	(4.4)	9.9	(4.4)
Enforcing temperature measurement	No	4245	28.8	(12.8)	9.1	(4.3)	10.2	(4.5)	9.4	(4.4)
	Yes	7737	30.6	(12.7)	9.8	(4.4)	11.0	(4.4)	9.9	(4.4)
Requesting that employees refrain from going to work when ill	No	2851	27.5	(13.1)	8.7	(4.5)	9.8	(4.6)	9.0	(4.5)
	Yes	9131	30.7	(12.5)	9.8	(4.4)	11.0	(4.4)	9.9	(4.3)

Abbreviations: IPC, infection prevention control; *SD*, standard deviation; UWES‐9, nine‐item version of the Utrecht Work Engagement Scale.

**TABLE 5:** Association between participants' work engagement and items of workplace IPC measures for COVID‐19

UWES‐9 items	Sex‐age adjusted			Multivariate		
Items of workplace IPC measures	Coef.	[95% CI]	*p*	Coef.	[95% CI]	*p*
Total score						
Stopping business trips; yes. (Ref., no)	0.33	[−0.30–0.95]	.305	0.40	[−0.22–1.01]	.209
Arranging health screenings for visitors; yes. (Ref., no)	0.43	[−0.16–1.02]	.153	0.50	[−0.08–1.09]	.089
Restricting work‐related social gatherings and entertainment; yes. (Ref., no)	0.79	[0.09–1.49]	.027	0.65	[−0.04–1.34]	.063
Restricting face‐to‐face meetings; yes. (Ref., no)	0.08	[−0.54–0.70]	.799	0.27	[−0.34–0.88]	.383
Encouraging mask‐wearing at work; yes. (Ref., no)	0.54	[−0.16–1.23]	.132	0.58	[−0.11–1.27]	.098
Installing partitions or changing the working environment; yes. (Ref., no)	0.59	[0.03–1.15]	.039	0.76	[0.20–1.32]	.007
Enforcing temperature measurement; yes. (Ref., no)	0.22	[−0.34–0.79]	.442	0.50	[−0.06–1.06]	.081
Requesting that employees refrain from going to work when ill; yes. (Ref., no)	1.85	[1.17–2.53]	<.001	2.01	[1.34–2.68]	<.001
Vigor						
Stopping business trips; yes. (Ref., no)	0.07	[−0.15–0.29]	.525	0.09	[−0.12–0.31]	.395
Arranging health screenings for visitors; yes. (Ref., no)	0.15	[−0.05–0.36]	.141	0.18	[−0.02–0.38]	.079
Restricting work‐related social gatherings and entertainment; yes. (Ref., no)	0.17	[−0.07–0.41]	.174	0.12	[−0.12–0.36]	.311
Restricting face‐to‐face meetings; yes. (Ref., no)	0.10	[−0.11–0.31]	.357	0.16	[−0.05–0.37]	.137
Encouraging mask‐wearing at work; yes. (Ref., no)	0.08	[−0.16–0.32]	.53	0.09	[−0.14–0.33]	.44
Installing partitions or changing the working environment; yes. (Ref., no)	0.20	[0.00–0.39]	.046	0.26	[0.06–0.45]	.009
Enforcing temperature measurement; yes. (Ref., no)	0.14	[−0.06–0.33]	.168	0.23	[0.03–0.42]	.021
Requesting that employees refrain from going to work when ill; yes. (Ref., no)	0.64	[0.41–0.88]	<.001	0.70	[0.46–0.93]	<.001
Dedication						
Stopping business trips; yes. (Ref., no)	0.14	[−0.08–0.36]	.207	0.17	[−0.05–0.39]	.123
Arranging health screenings for visitors; yes. (Ref., no)	0.08	[−0.12–0.29]	.429	0.11	[−0.09–0.32]	.288
Restricting work‐related social gatherings and entertainment; yes. (Ref., no)	0.35	[0.11–0.60]	.005	0.30	[0.06–0.54]	.014
Restricting face‐to‐face meetings; yes. (Ref., no)	−0.02	[−0.24–0.19]	.824	0.05	[−0.16–0.26]	.642
Encouraging mask‐wearing at work; yes. (Ref., no)	0.27	[0.02–0.51]	.034	0.27	[0.03–0.52]	.027
Installing partitions or changing the working environment; yes. (Ref., no)	0.23	[0.03–0.42]	.024	0.28	[0.09–0.48]	.004
Enforcing temperature measurement; yes. (Ref., no)	0.08	[−0.11–0.28]	.408	0.18	[−0.01–0.38]	.069
Requesting that employees refrain from going to work when ill; yes. (Ref., no)	0.70	[0.46–0.94]	<.001	0.76	[0.52–1.00]	<.001
Absorption						
Stopping business trips; yes. (Ref., no)	0.11	[−0.10–0.33]	.299	0.13	[−0.08–0.35]	.229
Arranging health screenings for visitors; yes. (Ref., no)	0.19	[−0.01–0.40]	.063	0.21	[0.01–0.42]	.038
Restricting work‐related social gatherings and entertainment; yes. (Ref., no)	0.27	[0.03–0.51]	.029	0.23	[−0.01–0.47]	.062
Restricting face‐to‐face meetings; yes. (Ref., no)	0.00	[−0.21–0.22]	.964	0.06	[−0.15–0.27]	.575
Encouraging mask‐wearing at work; yes. (Ref., no)	0.19	[−0.05–0.43]	.118	0.21	[−0.03–0.45]	.08
Installing partitions or changing the working environment; yes. (Ref., no)	0.17	[−0.03–0.36]	.095	0.22	[0.03–0.41]	.026
Enforcing temperature measurement; yes. (Ref., no)	0.00	[−0.20–0.20]	.999	0.09	[−0.11–0.28]	.375
Requesting that employees refrain from going to work when ill; yes. (Ref., no)	0.51	[0.27–0.74]	<.001	0.55	[0.32–0.79]	<.001

Abbreviations: IPC, infection prevention control; UWES‐9, nine‐item version of the Utrecht Work Engagement Scale; CI, confidence interval.

## 4 DISCUSSION

In this study, using the data from the CORoNaWork Project, we analyzed how the number of workplace IPC measures implemented in the workplace affected the participants' work engagement at their one‐year follow‐up. The results showed that there was an association between the implementation of workplace IPC measures and work engagement. In addition, we found that the more the workplace IPC measures were implemented, the higher the employees' work engagement was.

Since the COVID‐19 pandemic in Japan began in March 2020, the workplace policy regarding IPC measures was considered to have been established and relayed to the employees at the time of the baseline survey (December 2020). During the one‐year period between the baseline and the follow‐up survey, there were three waves of the COVID‐19 pandemic, during which we believe these IPC measures could have been implemented on a sustained basis. In addition, work engagement has been shown to be a sustained and general feeling, rather than a temporary and transient feeling toward work.^15^, ^16^, ^22^ Thus, we speculate that workplace IPC measures could have a sustained and generally positive effect on workers' mental health.

There are several reasons why proactive workplace IPC measures may result in high work engagement among workers. Certain studies have reported that anxiety and fear of COVID‐19 infection have directly led to negative mental health.^27^, ^28^, ^29^ In addition, we have reported that the more the workplace IPC measures are implemented in a workplace, the lower the psychological distress among workers.^30^ Workplace IPC measures may contribute to improved work engagement by decreasing employees' anxiety and mental stress. However, it is unclear whether these IPC measures are effective in reducing the risk of actual COVID‐19 infection; another study is needed to clarify this research question.

We found that the implementation of “Requesting that employees refrain from going to work when ill” has a strong influence on workers' work engagement. During an epidemic of contagious diseases, working in the workplace despite poor physical condition increases the risk of spreading the infection to colleagues and visitors.^31^ Therefore, during a COVID‐19 epidemic, those who have a fever, cold symptoms, or other health problems are strictly required to take leave from work according to the guidelines of the government and academic institutions.^8^ However, we speculated that guaranteed wages, health care assistance, and other forms of social support for employees are not available in all workplaces. Our previous study reported that people with less support from supervisors and coworkers are more likely to go to work when they are sick.^32^ Establishing workplace policies and labor procedures to prevent employees from going to work when sick might have positive psychological effects, such as reducing workers' fear of infection and increasing the perception of health support in the workplace and are a strong factor in increasing work engagement.

Workplace IPC measures can mainly be promoted using a top‐down process, that is, through a management system wherein actions are initiated at the highest level. It has been suggested that a strong top‐down process promotes a safe climate as well as workers' psychosocial safety in the workplace, and contributes to the reduction of mental distress among workers.^33^ A previous study has reported that during the COVID‐19 pandemic, the higher the workers' perceived workplace health support was, that is, the support for workers' lively working and healthy living provided by the workplace. The higher the health‐related quality of life was.^34^ In addition, clear policies surrounding workplace IPC measures have been reported to build trust between employers and workers.^35^ Actively promoting workplace IPC measures was also found to enhance corporate governance, increase employees' perceived workplace health support, and contribute to positive mental health, including increased work engagement.

Workplace IPC measures tend to be implemented more in secondary than in tertiary industries. Those who work for companies that implement more workplace IPC measures have been reported to be more well‐educated and belong to large‐sized companies.^26^, ^30^ These may result in various occupational factors, such as the difficulty in introducing workplace IPC measures in certain industries; the influence of risk awareness among management and employers; the presence or absence of interventions by occupational health specialists, such as occupational physicians and occupational hygienists; and the costs associated with implementing the countermeasures.

### 4.1 Limitations

This study has several limitations. First, the present study only included participants who were registered as Internet‐based survey monitors. Therefore, the sample of the study may not represent the general working population, and the generalizability of this study should be treated with caution. For example, there is a risk of overestimation if multiple participants belong to the same workplace. To deal with such issues, we made an effort to reduce sample bias by conducting random sampling stratified by gender and region of residence. Second, we did not consider the continuity or intensity of implementation of the workplace IPC measures. The baseline survey for this study was conducted in December 2020, when the third wave of the COVID‐19 pandemic was expanding nationwide. Therefore, we assumed that workplace IPC measures were implemented with high intensity. However, the intensity of the participants' self‐IPC measures and employees' perceptions thereof could differ between the period when the pandemic was under control and the period when the third wave was expanding. Third, because of the aforementioned reasons, it is difficult to conduct an analysis that takes into account changes in workplace IPC measures since the baseline. It is unclear at what point during the year changes in IPC measures occurred, and indeed, IPC measures have been thought to be constantly fluctuating due to directions such as state or semi‐state of emergency COVID‐19 measures by the government, and warnings regarding the COVID‐19 epidemic in each prefecture of Japan. However, we believe that policies concerning IPC measures for each workplace could be identified and established in the early stages of the COVID‐19 epidemic. In addition, we believe that the analysis method used in this study was more effective because the UWES‐9 used as the outcome variable is a long‐term stable psychological indicator.^15^, ^16^, ^22^ Forth, workplaces that implement many IPC measures may have ordinarily been engaged in health and productivity management, implementing workers' mental health measures, or concerned about the well‐being of their employees. Thus, the participants may have already had less mental distress at the baseline. However, it is difficult to evaluate this point in the present study.

## 5 CONCLUSIONS

This study found that promoting workplace IPC measures improved workers' work engagement. It was also shown that a dose–response relationship existed between workplace IPC measures and work engagement. Workplace IPC measures are expected to reduce workers' fear and anxiety related to COVID‐19 infection and to contribute to the mental health of workers. We believe that the implementation of workplace IPC measures could be important not only in controlling the COVID‐19 pandemic but also in promoting the positive mental health of workers.

## AUTHOR CONTRIBUTIONS

K.I. wrote the manuscript and analyzed the data; H.A. analyzed the data, and Y.F. was the chairperson of the study group. All the authors designed the research protocol and developed the questionnaire. All authors reviewed and approved the final manuscript.

## DISCLOSURE

Ethical approval: This study was approved by the ethics committee of the University of Occupational and Environmental Health, Japan (reference nos. R2‐079 and R3‐006). Informed consent: Informed consent from all participants was obtained via a website. Registry and registration no. of the study/trial: N/A. Animal studies: N/A.

## CONFLICT OF INTEREST

Authors declare no Conflict of Interests for this article.

## Data Availability

All data produced in the study are available upon reasonable request to the authors.
